# Indents

**Published:** 1918-10

**Authors:** 


### INDENTS

**☞Brief Articles of Technical or Scientific Value will receive notice and comment. Contributions invited.**

*Sensitive Cervical Surfaces* Apply a saturated solution of potassium carbonate in glycerine. This will be one ounce of potassium carbonate dissolved in two ounces of glycerine. Apply several times until sensitiveness disappears.—Dr. McClarty, in *Dental Digest.*

*Pulp Cappers* Place a thin piece of celluloid on a rubber block and press the round end of a large and warmed ball burnisher with a rotary motion into the celluloid to make a round cup shape. When cool remove and trim away all excess leaving a neat saucer shaped disk. It can be attached to a heated instrument as a means of carrying to place in the tooth cavity, or it may be attached to the instrument by a little warmed wax. Fill the disk with medicament desired and carry it to place over the exposed pulp and detach the instrument carefully. When the cap is settled to place cover with cement or other filling material. The celluloid is a good non-conductor and it will not stain the tooth or disintegrate. —Dr. F. L. Dungan, in *Digest.*

*Care of Handpiece* Place engine handpieces in a mixture of alcohol and liquid vaseline and leave over night. This will sterilize and lubricate the handpiece and keep it from rusting and wearing out so fast.—F. W. Frahm, in *Pacific Gazette.*

*Carving Amalgam Restorations* Pack the amalgam with considerable force and carve before it begins to set. It is liable to break away at the margins if it is carved after it begins to creak when cut. —Dr. Frahm, in *Pacific Gazette.*

*Services Rendered by Canadian Dental Corps*

Since July 15, 1915, when Canadian dentists began taking care of their soldiers teeth up to July 1, 1918, they have performed 1,821,966 operations of all kinds, from treatments to fillings and other restorations. This is certainly a tremendous contribution to the health and welfare of the soldiers. Who can estimate the invaluable assistance it has been in keeping up to its best the physical health of the soldiers and the additional power and resource it has given them?

*Gorgas Points to Good Coming From the War*

"The war will leave Americans in better physical condition than they have ever enjoyed before. Millions of soldiers and sailors will have had expert dental, medical, and surgical treatment and will come back from overseas lean and fit. The stay-at-homes will have profited by patriotic abstinance in eating.

"The nation will have attained its highest point in health. Already the army and navy are established upon the best health standards in the history of this or any other country. Where we had twenty-two deaths per thousand in the Spanish-American war we now have only three.

"To this standard the dentists of the country have contributed much. Clean, sound teeth, prevent much rheumatism, heart trouble, and diseases of the digestive organs. The dentists have rallied so well that we now have our full complement in the army with many more in training.

"The war, too, has caused great strides to be made in dentistry, surgery, and medicine. Many discoveries that will make the professions more valuable in times of peace have been made in portable relief stations or in base hospitals crowded with wounded soldiers. The stay-at-homes will profit by these discoveries after the war. One of the greatest developments has been the reconstruction of faces and here the dentists have taken an important part."—*Journal N. D. A.*

*Softening Bees Wax* Use a large quantity of water and have it only so warm that it will not blanch the wax. Allow the wax to remain in the water until it is softened entirely through the mass.—D. M. Show, in *Dental Record.*

*Army Dental Corps Appointments* No more appointments as officers are being made in the Army Dental Reserve Corps, excepting among those candidates who were examined prior to September 18, 1917, and who failed to meet the physical requirements and are now being given another opportunity by reason of the modification of the requirements since that time. The dental school at Camp Greenleaf, Fort Oglethorpe, Ga., is busily engaged in preparing both officers and enlisted men for their professional duties with the Army, particularly overseas. The course lasts two months, the first month being devoted to military instruction, special attention being given to cases emenating with the expeditionary force and from the forces of our allies. Eighty student-officers are selected each month for this instruction, the selections being made mostly from those already in the service, both in the regular dental corps and in the dental reserve corps. Enlisted men are sent to the school for a similar period of training, their course also consisting of a month each of military training and of professional subjects. Enlisted men are selected from dental students, and the instruction they receive at the school is for the purpose of fitting them as dental assistants.—*Army and Navy Register,* August 3.

*The Black Memorial* "G. V. Black, Father of Modern Dentistry. Born on the prairies of central Illinois; self educated, he became in his profession the foremost scientific investigator, writer and teacher of his time." This is the inscription on one of the tablets on the monument dedicated to Dr. Black, in Lincoln Park,

Chicago, by the National Dental Association, on August 8, 1918.

*Improvement In Dental Hygiene* This is not a nation of semi-invalids, for previous to the present war, this country had a lower death rate than any of the larger nations. Germany was second, but some distance behind this country. Relative to "ninety per cent. of the people having recourse to one of the many healing arts annually"—it is true that many people consult their physicians on the slightest pretext, such as a headache, backache or cold; but is it wise to condemn this practice in its entirety? It appears to me to show a desire, at least on the part of many, to have an early diagnosis of their ailments.

Our people are not going backward physically, morally and socially. Do not the successful campaigns carried on for improved sanitation, outdoor sleeping, clean alleys, better housing, a living wage, and against vice and alcoholism, indicate a better order of things to the essayist? They show that the world is getting to be a better place in which to live. Observe the efforts of the government to do away with vice and alcohol in the neighborhood of the cantonments recently established. Typhoid fever, at one time one of the greatest curses of the army, is now practically extinct in this branch of the service. During the period of the Spanish-American War, the government's oral hygiene activities were practically nil. Compare this with conditions in the present war, where dentists are assigned to both the Army and the Navy, and each enlisted man is given a tooth brush, and where a system is adopted whereby its proper use is insured. We are not going backward; we are improving.

Feeble, ill-directed, desultory efforts will not produce results when it comes to making an appeal to tax payers for funds to carry on school dental hygiene work.—Dr. Geo. F. Burke, in September *Journal N. D. A.* p. 915.

